# Airway-invasion-associated pulmonary computed tomography presentations characteristic of invasive pulmonary Aspergillosis in non-immunocompromised adults: a National Multicenter Retrospective Survey in China

**DOI:** 10.1186/s12931-020-01424-x

**Published:** 2020-07-07

**Authors:** Zhibo Liu, Yuping Li, Xinlun Tian, Qinghua Liu, Erran Li, Xiaoying Gu, Min Liu, Jiuyang Xu, Zhiyi He, Yi Huang, Shuyun Xu, Guoxiang Lai, Yusheng Chen, Xiangyan Zhang, Tiantuo Zhang, Jinfu Xu, Lanyan Zhu, Jieming Qu, Bin Cao

**Affiliations:** 1 Department of Respiratory and Critical Care Medicine, Clinical Microbiology and Infectious Disease Lab, China-Japan Friendship Hospital, Beijing, 100029 China; 2 Department of Respiratory and Critical Care Medicine, The First Affiliated Hospital of Wenzhou Medical University, Wenzhou, China; 3 Department of Respiratory Medicine, Peking Union Medical College Hospital, Chinese Academy of Medical Sciences and Peking Union Medical College, Beijing, China; 4 Department of Respiratory Medicine, Shanghai East Hospital, Tongji University School of Medicine, Shanghai, China; 5 Institute of Respiratory Disease, The First Affiliated Hospital of China Medical University, Shenyang, China; 6 Department of Institute of Clinical Medical Sciences, China-Japan Friendship Hospital, Beijing, China; 7 The department of radiology, China-Japan Friendship Hospital, Beijing, China; 8 Tsinghua University School of Medicine, Beijing, China; 9 Department of Respiratory Medicine, The First Affiliated Hospital of Guangxi Medical University, Nanning, China; 10 Department of Respiratory and Critical Care Medicine, Changhai Hospital, the Second Military Medical University, Shanghai, China; 11 Department of Respiratory and Critical Care Medicine, Key Laboratory of Pulmonary Diseases of Health Ministry, Tongji Hospital, Tongji Medical College, Huazhong University of Science and Technology, Wuhan, China; 12 Department of Respiratory and Critical Care Medicine, Fuzhou General Hospital of Fujian Medical University, Fuzhou, China; 13 Department of Respiratory and Critical Care Medicine, Fujian Provincial Hospital, Provincial Clinical Medical College, Fujian Medical University, Fuzhou, China; 14 Department of Respiratory and Critical Care Medicine, Guizhou Provincial People’s Hospital, Guiyang, Guizhou Province China; 15 Department of Pulmonary and Critical Care Medicine, Third Affiliated Hospital of Sun Yat-sen University, Institute of Respiratory Diseases of Sun Yat-Sen University, Guangzhou, China; 16 Department of Respiratory and Critical Care Medicine, Shanghai Pulmonary Hospital, Tongji University School of Medicine, Shanghai, China; 17 Department of Respiratory Medicine, The Second Xiangya Hospital, Central-South University, Changsha, China; 18 Department of Respiratory and Critical Care Medicine, Ruijin Hospital, School of Medicine, Shanghai Jiaotong University, Shanghai, 200025 China

**Keywords:** Invasive pulmonary aspergillosis, Non-immunocompromised, Airway-invasive, Early diagnosis, National multicenter retrospective survey

## Abstract

**Background:**

The European Organization for Research and Treatment of Cancer/Mycoses Study Group (EORTC/MSG) criteria are widely used in the diagnosis of invasive pulmonary aspergillosis (IPA), but they only apply to immunocompromised patients. We here aimed to identify clinical characteristics helpful to the diagnosis of IPA in non-immunocompromised patients.

**Methods:**

This is a multicenter retrospective study. Data were collected from adult patients with IPA admitted to 15 **tertiary hospitals in China** from 2010 to 2016.

**Results:**

We included 254 patients in the study, of whom 66 (26.0%) were immunocompromised, and 188 (74.0%) were not. Airway-invasion-associated computed tomography (CT) signs including patchy exudation along the airway (67.6% vs. 45.5%, *P* = 0.001) and thickened airway wall (42.0% vs. 16.7%, *P* < 0.001) were more common in non-immunocompromised patients than in immunocompromised ones, and angio-invasive CT signs were more common in immunocompromised patients (55.3% vs.72.7%, *P* = 0.013). Typical angio-invasive CT signs were delayed in non-immunocompromised IPA patients, whereas airway-invasive signs appear earlier. Host immunocompromised condition was associated with ICU admission and/or intubation (OR 1.095; 95% CI 1.461–6.122; *P* = 0.003). Poor prognosis (35.5% vs. 21.1%, *P* = 0.005) was more common in immunocompromised patients.

**Conclusion:**

Airway-invasion-associated CT presentations at early stages of the disease are characteristic of IPA in non-immunocompromised hosts.

## Introduction

Invasive pulmonary aspergillosis (IPA) is a life-threatening infectious disease, but its diagnosis is still challenging [1, 2]. Pulmonary histopathological examination remains the gold standard for IPA diagnosis, but the fact that many patients do not tolerate tissue biopsy has prevented wide clinical usage. Several clinical diagnostic criteria have been raised for IPA. Among them, the European Organization for Research and Treatment of Cancer/Invasive Fungal Infections Cooperative Group and the National Institute of Allergy and Infectious Diseases Mycoses Study Group (EORTC/MSG) criteria are the most widely used [1]. The EORTC/MSG criteria include host factors (mainly immunocompromised conditions) among pre-requisites for a possible IPA diagnosis, since, traditionally, non-immunocompromised patients are considered “non-classic” or “non-traditional” hosts for IPA [3, 4]. This makes early diagnosis of IPA in non-immunocompromised patients difficult when microbiological and histological tests were not performed.

Recently, some underlying diseases irrelevant to the host immune status, such as chronic obstructive pulmonary disease (COPD), diabetes, and pre-influenza infection, have been suggested as IPA risk factors [5–7]. These emerging findings have broadened our understanding of IPA host factors, but they have not been accepted into the diagnostic criteria, and IPA in non-immunocompromised patients is still not well-defined. For example, in the current criteria, the description of radiological findings is imperfect. The EORTC/MSG criteria require one of three CT presentations for the vascular invasion as a prerequisite for diagnosing IPA: well-circumscribed lesions with or without a halo sign, air-crescent sign, and cavity. These criteria have relatively high specificity, but they are very strict, which makes them unsuitable for non-immunocompromised IPA. Previous studies have established that the characteristic halo sign and air-crescentic sign only present in 60 and 10% IPA patients, respectively [8]. Some airway-invasion CT signs, like nodules and patchiness, are often seen in IPA [9, 10], but they were previously described as “non-specific radiological findings” [11]. However, in IPA definitions of COPD and influenza patients, the inclusion requirements of radiological images are broadly discussed. CT presentations in non-immunocompromised patients remain unclear.

The EORTC/MSG criteria have been widely accepted for use in immunocompromised IPA patients. In contrast, understandings of IPA in non-immunocompromised patients are still poor. Immunocompromised IPA can be recognized, but the diagnosis of non-immunocompromised IPA remains challenging. In this retrospective multicenter study, we compared clinical characteristics between immunocompromised and non-immunocompromised patients and identified some clinical characteristics that would be easy to use, facilitating the diagnosis of IPA.

## Methods

### Study participants and design

We performed a retrospective multicenter survey of 15 major tertiary hospitals. These hospitals were distributed throughout the country (11 cities across northern, southern, southeastern, northeastern, and southwestern China).

All included patients were over 18 years old. They were admitted from January 2010 to December 2016 and received chest CT scans. The inclusion criteria differed from the current IPA criteria, including EORTC/MSG definition, Bulpa criteria, and modified definition of IPA in influenza patients. Bulpa criteria are used for COPD patients. The modified definition of IPA in influenza patients was based on the presence of clinical, radiological, and mycological criteria in influenza cohort. Similar to these two definitions, this definition of inclusion criteria did not require risk factors typical of immunocompromised patients.

The diagnosis of IPA was based on affirmative clinical criteria and objective evidence of the presence of *Aspergillus* (Table 1). Patients who were diagnosed with other types of pulmonary aspergillosis but not IPA, including chronic pulmonary aspergillosis (CPA) and allergic bronchopulmonary aspergillosis (ABPA), were excluded.

**Table 1:** **The inclusion criteria of IPA**

Meet the conditions of both clinical criteria and objective evidence of ***Aspergillus***	
Clinical criteria:1+ 2 and/or 3, <1 month from onset	
1. signs or symptoms in recent one month, the presence of at least 1 of the following:	
a. Fever, refractory to at least 3 days or recrudescent fever after a period of defeverescence in spite of appropriate antibiotic therapy	
b. dyspnea	
c. Haemoptysis	
d. Pleural friction rub or chest pain	
e. Worsening respiratory insufficiency in spite of appropriate antibiotic therapy and ventilatory support	
2. Radiological criteria, the presence of at least 1 of the following:	
a. Imaging signs presented in EORTC/MSG criteria: dense, well-circumscribed lesions with or without a halo sign; air-crescent sign; cavity	
b. Any other infiltrate on pulmonary imaging	
3. Bronchoscopy: Tracheobronchial ulceration, nodule, pseudomembrane, plaque, or eschar seen on bronchoscopy analysis	
Objective evidence of *Aspergillus*, the presence of at least 1 of the following:	
1. A positive *Aspergillus* culture from low respiratory tract	
2. A GM optical index on BALF of ≥1	
3. A GM optical index on serum of ≥0.5	
4. Recovery of *Aspergillus* by culture of tissue or sterile material	
5. Histopathologic examination of a specimen obtained by biopsy in which *Aspergillus* hyphae were seen accompanied by evidence of associated tissue damage	

*GM* Galactomannan

*BALF* Bronchoalveolar lavage fluid

The cohort was divided into patients with and without immunocompromised host factors.

Ethical approval was obtained from the institutional review board (No. 2017–205). Informed consent was waived due to the retrospective nature of the study.

### Definitions

Immunocompromised conditions included the following: recent history of neutropenia (< 0.5 × 10^9^ /L); treatment with corticosteroids (at a mean minimum dose of 0.3 mg/kg/day of prednisone equivalent for > 3 weeks, or equivalent cumulative doses) or other immunosuppressants (during the past 3 months); chemotherapy during the past 6 months or chest radiotherapy during the past 1 month; allogeneic stem cell transplant during the past 1 year or solid organ transplant at any time; inherited severe immunodeficiency [1].

According to the EORTC/MSG definition, proven IPA criteria include host factors, clinical features, and histopathological evidence. Probable criteria include host factors, clinical features, and microbiological evidence. Possible criteria include only host factors and clinical features.

All 254 patients underwent chest CT scans. Only the initial CT was included for analysis if the patient received more than one CT scan. Angio-invasive CT presentations include dense, well-circumscribed lesion(s) with or without a halo sign, cavity or air crescent sign, wedge-shaped consolidation, and internal low-attenuated lesion [1, 12]. Centrilobular nodule, patchy distribution along the airway, tree-in-bud sign, and thickened tracheal wall were considered signs of airway invasion [11]. Two pulmonary physicians independently reviewed the chest CT scans, and one experienced chest radiologist adjudicated any difference in interpretation between the two primary reviewers.

The time of diagnosis here refers to days from the onset of symptoms to diagnosis.

Poor prognosis included death and withdrawal of life support, which was performed when critically ill patients were unlikely to make a meaningful recovery. Life support was only withdrawn with the consent of the patient’s relatives.

### Statistical analysis

All of the analyses were performed using SPSS 22.0 software. Normally distributed continuous variables are here expressed as x ± SD and compared using a t test. Nonnormally distributed continuous variables are here expressed as median and quartiles and compared using a Wilcoxon rank-sum test. Categorical variables were compared using the χ2 test. *P* values< 0.05 were considered significant. For the entire population of IPA patients, univariate and multivariate analysis with binary logistic regression was used to identify independent risk factors for ICU and mechanical ventilation. To assess which factor was independently associated with angio-invasive CT signs, a binary logistic regression analysis was also performed (on the population of patients with angio-invasive CT signs). Univariate logistic regression analysis was used to screen risk factors (*P* < 0.1), then the selected ones were analyzed by multivariate regression analysis (*P* < 0.05).

## Results

### Patients

**Fig. 1:**
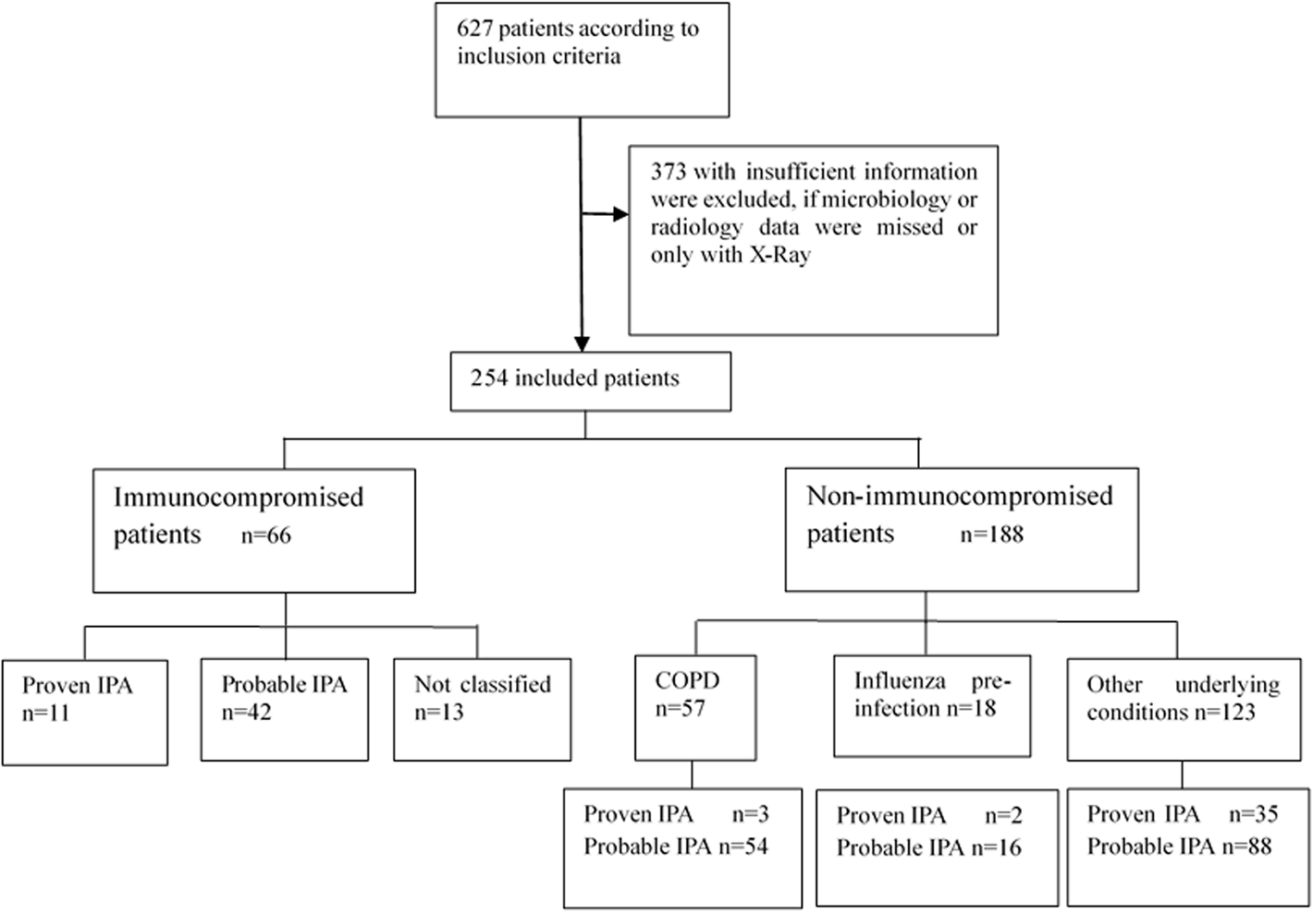
Classification of the inclusion patients by host factors

We here included 672 patients who were treated at 15 tertiary comprehensive hospitals from 11 cities in China between January 1, 2010, and December 31, 2016. We excluded 373 because of insufficient clinical or radiological data. Patients who underwent X-ray but no CT scans were also excluded. In total, 254 patients were included in the final analysis (Fig. 1). Their host factors and underlying conditions are listed in Supplementary-Table. Immunocompromised conditions were found in 66 patients, the most common being receiving corticosteroid treatment (46, 18.1%) and immunosuppressive agents (31, 12.2%). Among them, 13 patients did not present with angio-invasive CT signs and did not meet EORTC/MSG criteria, so they were considered “not classified” cases. There were 36 (14.2%) patients who had no underlying conditions.

Patients with confirmed IPA (51, 20.1%) had various kinds of underlying conditions. Among them, 40 were not immunocompromised.

### Characteristics of immunocompromised and non-immunocompromised patients

Characteristics of immunocompromised and non-immunocompromised patients are summarized in Table 2.

**Table 2:** Characteristics of immunocompromised and non- immunocompromised IPA patients

	IPA patients (*n* = 254)	Immunocompromised (*n* = 66)	non- immunocompromised (*n* = 188)	*p* value
**Demographics**				
Sex, male (%)	179 (70.5%)	42 (72.9%)	137 (63.6%)	0.157
Age, yrs.	61 ± 15	54 ± 15	64 ± 15	< 0.001^*^
**Admission data**				
Diagnostic time, days (IQR)	20 (12 ~ 30)	18 (10 ~ 36)	20 (12 ~ 30)	0.461
Mechanical ventilation	51 (20.1%)	18 (27.1%)	33 (17.6%)	0.09
ICU admission	62 (24.4%)	22 (33.3%)	40 (21.3%)	0.05
Poor prognosis	57 (22.4%)	23 (34.8%)	34 (18.1%)	0.005^*^
**Symptoms**				
fever	188 (74.0%)	44 (66.7%)	144 (76.6%)	0.114
productive cough	226 (89.0%)	53 (80.3%)	173 (92.0%)	0.009^*^
hemoptysis	38 (15.0%)	8 (12.1%)	30 (16.0%)	0.452
dyspnea	158 (62.2%)	37 (56.1%)	121 (64.4%)	0.231
chest pain	15 (5.9%)	3 (4.5%)	12 (6.4%)	0.586
**Diagnostics**				
Histopathology	46	10	36	
Lung tissue culture of positive	29	1	28	
Culture of LRT preformed	254	66	188	
LRT culture positive	196 (77.1%)	50 (75.7%)	146 (77.7%)	0.553
Serum GM preformed	107	19	88	
Serum GM positive	59 (55.1%)	12 (63.1%)	47 (53.4%)	0.388
BALF GM preformed	38	7	31	
BALF GM positive	36 (94.7%)	7 (100%)	29 (93.4%)	0.490

*GM* Galactomannan

*BALF* Bronchoalveolar lavage fluid

*LRT* Low respiratory tract

note: * *p*<0.05

IPA was diagnosed at a median of 20 days after onset of disease (IQR 12–30 days). Among 254 patients, 196 had positive low respiratory tract culture. Bronchoalveolar lavage fluid (BALF) galactomannan test was performed in 38 patients, of whom 36 received positive results. Serum galactomannan tests were performed in 107 patients, and results were only positive in 59 of them.

Patients who need ICU admission (21.3% vs.33.3%, *P* = 0.05) and intubation for mechanical ventilation (17.6% vs.27.1%, *P* = 0.09) were fewer in non-immunocompromised patients. The poor prognosis was more common in immunocompromised patients (35.5% vs. 21.1%, *P* = 0.005).

Immunocompromised conditions (OR 3.108; 95% CI 1.524–6.339; *P* = 0.002), age (OR 1.033; 95% CI 1.009–1.058; *P* = 0.008), and dyspnea (OR 3.484; 95% CI 1.615–7.515; *P* = 0.001) were independently associated with ICU admission and intubation.

### CT presentations in IPA patients

Chest CT presentations in immunocompromised and non-immunocompromised patients are described in Table 3, and images are shown in the Supplementary Figure.

**Table 3:** CT presentations of immunocompromised and non-immunocompromised IPA patients

	IPA patients (*n* = 254)	Immunocompromised (*n* = 66)	Non-immunocompromised (*n* = 188)	***p*** value
**Angio-invasive signs**				
cavity	102 (40.2%)	31 (47%)	71 (37.8%)	0.189
air crescent sign	29 (11.4%)	9 (13.6%)	20 (10.6%)	0.510
soft-tissue mass	80 (31.5%)	24 (36.4%)	56 (29.8%)	0.322
wedge shaped consolidation	25 (9.8%)	11 (16.7%)	14 (7.4%)	0.031^*^
halo sign	52 (20.5%)	17 (25.8%)	35 (18.6%)	0.216
**Airway invasive signs**				
centrilobular nodules	95 (37.4%)	25 (37.9%)	70 (37.2%)	0.926
patches distributing along the airway	157 (61.8%)	30 (45.5%)	127 (67.6%)	0.001^*^
airway wall thickness	90 (35.4%)	11 (16.7%)	79 (42.0%)	< 0.001^*^
tree in bud sign	93 (36.6%)	18 (27.3%)	75 (39.9%)	0.067
pleural effusion	70 (27.6%)	17 (25.8%)	53 (28.2%)	0.703
atelectasis	15 (5.9%)	3 (4.5%)	12 (6.4%)	0.586

*EORTC/MSG* European Organization for Research and Treatment of Cancer/Invasive Fungal Infections Cooperative Group and the National Institute of Allergy and Infectious Diseases Mycoses Study Group

note: * *p*<0.05

Of all 254 patients receiving chest CT scans, angio-invasive signs were presented in 152 (59.8%) patients, including cavity (40.2%), air crescent sign (11.4%), soft-tissue mass (31.5%), wedge-shaped consolidation (9.8%), and halo sign (20.5%). Other signs included centrilobular nodules (37.4%), patches distributing along the airway (61.8%), thickened airway wall (35.4%), tree-in-bud sign (36.6%), pleural lesions (27.6%), and atelectasis (5.9%). In 42.1% patients, Angio-invasive signs and other signs presented at the same time.

Angio-invasive signs were more common in immunocompromised patients (72.7% vs. 55.3%, *P* = 0.013). Airway-invasion-associated CT signs were more common in non-immunocompromised patients (85.6% vs. 65.2%, *P* < 0.001). Cavity (47%) and patches distributing along the airway (45.5%) were the most common signs in immunocompromised patients, whereas patchy (67.6%) and thickened airway wall (42.0%) were common features in non-immunocompromised patients.

Chest CT scans were taken during hospitalization. The median time from illness onset to the first CT examination was 17 days (IQR 9.25–29.25 days). By quartile, we set day 10 as time points of the early stage of the disease. As of the 10th day, 75 patients had undergone chest CT, and angio-invasive signs and airway-invasive signs were seen in 36 (48.0%) and 60 (80.0%) patients, respectively. Halo sign was significantly more common in immunocompromised patients (30.0% vs. 8.9%, *P* = 0.018). Among non-immunocompromised patients, patchiness along the airway (68.9% vs. 40.0%, *P* = 0.013) and airway thickness (48.9% vs. 10.0%, *P* < 0.001) were more common. Air crescent sign was rare (4.4% vs. 6.7%, *P* = 0.675).

**Fig. 2:**
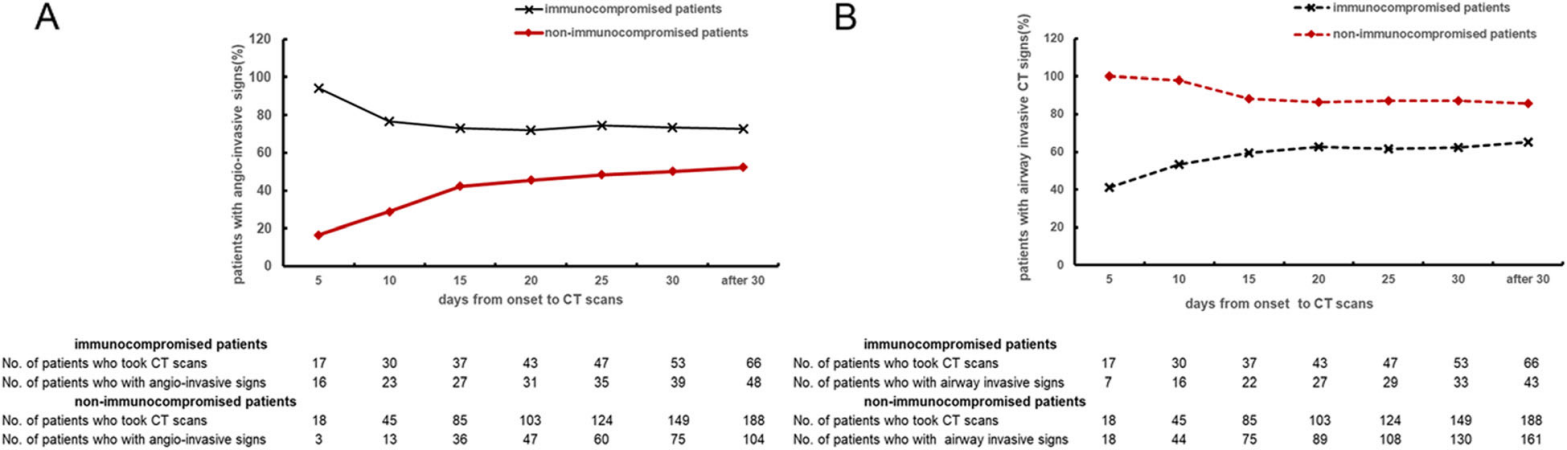
Trends in radiological patterns in patients who underwent chest CT at different times. A. The percentage of angio-invasive CT signs in immunocompromised and non-immunocompromised patients. B. The percentage of airway-invasive-associated CT signs in immunocompromised and non-immunocompromised patients

Figure 2 shows the dynamic change in radiology patterns in IPA patients. Among non-immunocompromised patients, angio-invasive signs were only seen in 28.9% during the early stage, but it became more common as the disease progressed. Conversely, almost all patients presented with airway-invasive signs at the beginning of the disease.

To assess which factor was independently associated with angio-invasive signs, a logistic regression analysis was performed. This analysis confirmed an independent association between time from onset to CT examination and angio-invasive signs (OR 1.034; 95% CI 1.001–1.058; *P* = 0.004). Other variables included in the multivariate analysis were age, use of corticosteroids, use of immunosuppressive agent, diabetes, liver dysfunction, renal insufficiency, and COPD. Patients with increased age (OR 0.973; 95% CI 0.952–0.995; *P* = 0.016) and COPD (OR 0.468; 95% CI 0.231–0.946; *P* = 0.035) were less likely to present angio-invasive signs.

## Discussion

In this study, we presented underlying conditions and radiological findings in 254 IPA patients and compared clinical characteristics, especially CT presentations, between immunocompromised and non-immunocompromised patients. We found only 26% patients had immunocompromised conditions. Airway-invasion-associated CT presentations may be predictive signs for identifying IPA, before microbiology and histology evidence are taken into consideration.

IPA was first recognized in patients with underlying conditions of neutropenia and immunosuppression [3]. In recent years, emerging studies found other mechanisms, including injury to the airway barrier, also increased susceptibility to IPA [13, 14]. In this way, immunosuppression ceased to be a pre-requisite for IPA, and strict interpretation of the immunocompromised conditions for IPA increased the risk of misdiagnosis [15]. The significance of the EORTC/MSG criteria was the classification of IPA diagnosis into three levels: proven, probable, and possible. For an immunocompromised host with typical CT presentations, even if there was no microbiological or histological evidence of *Aspergillus*, the patient is very likely to be diagnosed with possible IPA, and antifungal treatment is immediately given. However, among non-immunocompromised patients, diagnosis of IPA cannot be made without microbiological or histological evidence, because the immunocompromised host factors and angio-invasive CT signs are often present.

Airway-invasive signs (airway infiltration including patchiness along the airway, thickened airway wall, and tree-in-bud signs) have traditionally been considered non-specific signs of IPA [16, 17]. However, emerging reports have shown that airway-invasive signs were more pronounced in non-immunocompromised patients [10, 18]. Airway-invasion-associated CT presentations are not specific to IPA. For example, tree-in-bud sign is also seen in tuberculosis patients, and centrilobular nodules can also be seen in mycoplasma pneumonia. CT presentations provide clues, but the diagnosis requires a comprehensive evaluation.

We hypothesize that airway-invasive and angio-invasive signs are successive phases of IPA. *Aspergillus invades the lung through inhalation.* Nucci et al. reported that more severe neutropenia in the IPA host was associated with faster angioinvasion, leading to a very short early airway-invasion phase [11, 19]. According to a previous study, about 80% of patients met EORTC/MSG radiological criteria within 4 weeks in a cohort with hematologic diseases [20]. However, in non-immunocompromised patients, angio-invasive signs were seen in only half of the cases over the course of 4 weeks (Fig. 2a). This indicated that in non-immunocompromised IPA patients, angio-invasive signs may be delayed. Airway invasion may be a more advisable means of identifying non-immunocompromised IPA patients at the early stage. Some patients never develop angio-invasive CT presentations. Discontinuing the use of immunosuppressive drugs and initiation of anti-fungal drugs may delay angio-invasion. With the improvement of host immunity and early treatment, exacerbation of IPA may be avoided.

The present work has some limitations. First, because only IPA patients with CT examinations were included, the total number of cases was relatively small. Second, because this was a retrospective survey, some clinical information was unavailable, such as body-mass index, CD4 and CD8 counts, and PaO_2_/FiO_2_. The time of CT examination was also irregular. Here, we only describe CT presentations of populations in different stages during the course of IPA course, but we could not observe radiological signs longitudinally. To explore the evolution of CT presentations, especially in non-immunocompromised patients, further prospective studies are needed.

The current EORTC/MSG diagnostic criteria for IPA are not perfect. Immunocompromised IPA patients are more easily identified, but it is difficult to form an early diagnosis of non-immunocompromised patients. Typical angio-invasive CT signs were delayed in non-immunocompromised IPA patients, whereas airway-invasive signs appear earlier. When an acute pulmonary infection patient occurs alongside airway-invasion signs in chest CT, IPA should be considered, regardless of the patient’s underlying condition.

## Supplementary information

**Additional file 1: Table S1.** Underlying conditions of IPA patients. **Figure S1.** Radiological presentations of IPA patients.

## Data Availability

All data generated or analyzed during this study are included in this published article.

## Abbreviations

IPA: Invasive pulmonary aspergillosis
EORTC/MSG: European organization for research and treatment of cancer/invasive fungal infections cooperative group and the national institute of allergy and infectious diseases mycoses study group
COPD: Chronic obstructive pulmonary disease
CPA: Chronic pulmonary aspergillosis
ABPA: Allergic bronchopulmonary aspergillosis
