# Supplementary material for: Airway-invasion-associated pulmonary computed tomography presentations characteristic of invasive pulmonary Aspergillosis in non-immunocompromised adults: a National Multicenter Retrospective Survey in China
Source: Respir Res. 2020 Jul 7;21:173. doi: 10.1186/s12931-020-01424-x (PMC7341597; doi:10.1186/s12931-020-01424-x)
Supplement: Supplementary file 1 — Additional file 1: Table S1. Underlying conditions of IPA patients. Figure S1. Radiological presentations of IPA patients. [file 12931_2020_1424_MOESM1_ESM.zip › s-table.docx.pdf]

S-table. Underlying conditions of IPA patients

Underlying conditions	All patients n=254	Proven n=51	Others n=203
immunocompromised host factors	66 (26.0%)	11 (21.6%)	55 (27.1%)
use of corticosteroid	46 (18.1%)	7 (13.7%)	39 (19.2%)
use of immunosuppressive agent	31 (12.2%)	4 (7.8%)	27 (13.3%)
chemotherapy	12 (4.7%)	3 (5.9%)	9 (4.4%)
solid organ transplantation	5 (2.0%)	3 (5.9%)	2 (1.0%)
neutropenia	7 (2.8%)	2 (3.9%)	5 (2.5%)
Non-immunocompromised host factors	188 (74.0%)	40 (78.4%)	148 (72.9%)
COPD	57 (22.4%)	3 (5.9%)	54 (26.6%)
diabetes	45 (17.7%)	15 (29.4%)	30 (14.8%)
viral pre-infection	18 (7.1%)	2 (3.9%)	16 (7.9%)
liver dysfunction	17 (6.7%)	6 (11.8%)	11 (5.4%)
bronchiectasis	6 (2.4%)	2 (3.9%)	4 (2.0%)
asthma	11 (4.3%)	3 (5.9%)	8 (4.0%)
renal insufficiency	9 (3.5%)	1 (2.0%)	8 (4.0%)
Without underlying diseases	36 (14.3%)	8 (15.7%)	24 (11.8%)

COPD: chronic obstructive pulmonary disease

EORTC/MSG: European Organization for Research and Treatment of Cancer/Invasive Fungal Infections Cooperative Group and the National Institute of Allergy and Infectious Diseases Mycoses Study Group

S-Figure. Radiological presentations of IPA patients

A. cavity (grey arrow) and air crescent sign (black arrow)
B. Halo sign (grey arrow)
C. air crescent sign (grey arrow)
D. wedge shaped consolidation (black arrow)
E. patches distributing along the airway of right lower lobe bronchus (grey arrow)
F. tree in bud sign (grey arrow)
G. centrilobular nodules (black arrow)
H. atelectasis (grey arrow) and airway wall thickness (black arrow)
