# Supplementary figures and images for: Airway-invasion-associated pulmonary computed tomography presentations characteristic of invasive pulmonary Aspergillosis in non-immunocompromised adults: a National Multicenter Retrospective Survey in China
Source: Respir Res. 2020 Jul 7;21:173. doi: 10.1186/s12931-020-01424-x (PMC7341597; doi:10.1186/s12931-020-01424-x)

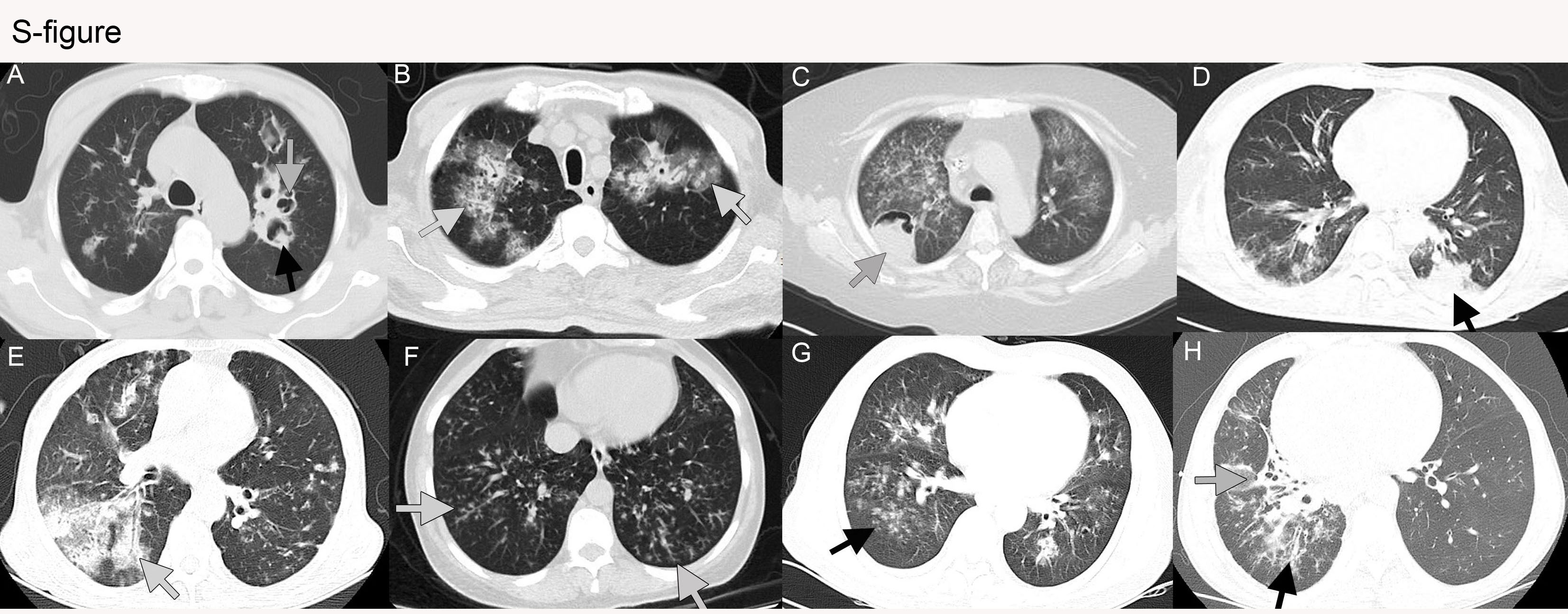

Supplement: Supplementary file 1 — Additional file 1: Table S1. Underlying conditions of IPA patients. Figure S1. Radiological presentations of IPA patients. [file 12931_2020_1424_MOESM1_ESM.zip › s-FIGURE.jpg]
